# Cerebral Brain Abscess Mimicking Psychiatric Presentation in an HIV-Positive Patient With Post-traumatic Stress Disorder and Bipolar Disorder: A Case Report

**DOI:** 10.7759/cureus.72279

**Published:** 2024-10-24

**Authors:** Yiorgos Antoniadis, Augustine Umeozor, Abdul Mohit

**Affiliations:** 1 College of Medicine, St. George's University School of Medicine, Saint George, GRD; 2 Behavioral Health, Kings County Hospital Center, New York, USA

**Keywords:** bipolar disorder (bpd), catatonia, clinical psychiatry neurology, immunocompromised brain abscess, neuroimaging, post traumatic stress disorder (ptsd), ventriculoperitoneal shunts

## Abstract

Cerebral brain abscesses, though relatively rare, present significant clinical challenges with high morbidity and mortality rates, especially in immunocompromised patients. These abscesses can mimic psychiatric disorders due to their space-occupying effects, complicating diagnosis and treatment. This case report details a 47-year-old HIV-positive African American woman with a history of bipolar disorder, post-traumatic stress disorder (PTSD), and anxiety who presented to the psychiatric emergency department with altered mental status, catatonia, and disorganized behavior. Initial assessment was challenging due to her psychiatric history and unusual symptoms, including catatonia and disorganized behavior. Laboratory findings indicated acute kidney injury and hypernatremia. Concerns about increased intracranial pressure led to imaging, revealing a large left frontal lobe mass with significant edema and midline shift, suggestive of a cerebral abscess with obstructive hydrocephalus. The patient underwent emergent intubation, surgical aspiration of the abscess, ventricular-peritoneal shunt placement, and left hemicraniectomy, with cultures identifying *Streptococcus viridans*. Post-operatively, she showed gradual improvement in mental status and motor function, ultimately being discharged to a rehabilitation facility. This case underscores the importance of considering organic etiologies, including brain abscesses, in immunocompromised patients with new or atypical psychiatric presentations. Comprehensive diagnostic assessments and neuroimaging are crucial for accurate diagnosis and timely intervention, with multidisciplinary approaches essential for optimal management and improved patient outcomes.

## Introduction

Cerebral brain abscesses, though relatively rare, can present with a variety of complicated clinical challenges and carries a high morbidity and mortality rate. Brain abscesses are usually defined by localized infections within the brain parenchyma and can display a variety of symptoms, contingent on the size, location, and immunocompromised state of the patient [1]. Headache, fever, and focal neurological impairments are examples of traditional symptoms associated with brain abscesses; however, potential psychiatric symptoms may manifest due to the space-occupying nature of the lesion. The effect on brain function may influence behavior and cause neuropsychiatric changes that can mimic symptoms of primary neurological or psychiatric disorders. An example of this is catatonia, a neuropsychiatric syndrome that is characterized by abnormal movements, behaviors, and akinesia [2]. Although catatonia is more commonly recognized as a product of worsening primary psychiatric conditions, it can also be a symptom of organic disorders such as brain tumors and, less commonly, cerebral brain abscesses. In such presentations, the utilization of neuroimaging provides an effective means of confirming an organic cause is present. It thus may help develop a differential diagnosis that includes neurological conditions. In this case study, we report on an HIV-positive patient with a past psychiatric history who developed catatonia and deteriorating altered mental status due to a large space occupying the left frontal cerebral abscess.

## Case presentation

A 47-year-old HIV-positive African American woman with a longstanding psychiatric history of bipolar disorder, post-traumatic stress disorder (PTSD), and anxiety presented to the psychiatric emergency department (ED) on the morning of March 8, 2024, with altered mental status, catatonia, and disorganized behavior. She was brought in by the New York Police Department (NYPD) for a psychiatric evaluation after failing to attend an outpatient appointment (March 6, 2024) with her psychiatrist. When the psychiatrist contacted the patient’s residence due to the missed appointment, the patient was disoriented, bizarre, and confused over the phone. She noticed a change and a new onset of hoarseness in her voice when speaking to her. She was disoriented to person, place, time, and situation. This warranted a crisis team (mobile crisis unit) visit to her residence, in which the crisis team noted she was disorganized and malodorous upon entering with blood-soaked underwear, and she was not adherent to medications. Documentation and reports by family suggest continued compliance with medications up to this point with no adverse effects, and the patient always kept a clean home and was generally pleasant while living alone. Her son had mentioned that the patient had been struggling with PTSD symptoms weeks prior to the emergency room visit, with increasing nightmares, anxiety, and hypervigilance. Leading up to the encounter, the patient felt frustrated with these symptoms and felt the PTSD “had a grasp on her”. Her son denies worsening of manic or psychotic symptoms, hallucinations, suicidal/homicidal ideations, or worsening of depressive symptoms, which aligns with the patient’s reported documentation. The patient was receiving clonazepam 0.5 mg as needed for panic attacks and quetiapine 100 mg orally in the a.m. and 300 mg orally at bedtime.

Upon entering the ED, the patient was mute but cooperative during triage. She seemed internally preoccupied and slightly disorganized, often looking over her right shoulder towards no clear stimuli, and was unable to fully participate in her H&P. When further attempts were made to engage her, she did not make eye contact, did not produce any spontaneous movements, and was not responding to questions. Initially, the primary concern was disorganized behavior; however, in psychiatric ED, the patient was also exhibiting a state of catatonia and altered mental status, which she has never presented with in her past psychiatric history. This was further complicated by the patient's mutism, self-urination, decreased responsiveness, rigidity, and new onset tremulousness. The acute state of the patient necessitated urgent neuroimaging and management. As a result, the Bush-Francis Catatonia Rating Scale (BFCRS) was not used to measure the severity of catatonia at the time. The BFCRS was retrospectively calculated to be 14. An abnormal increase in creatinine (1.40 mg/dL) and sodium (163 mmol/L) levels on laboratory findings resulted in a further diagnosis of acute kidney injury (AKI) and hypernatremia, which ultimately led to a transfer from the psychiatric ED to the medical emergency department (Tables 1, 2). Six hours later, in the ED, the patient was even less responsive to interaction and stimuli and developed new hypertension with widened pulse pressure and bradycardia (Table 3). This was concerning for Cushing’s triad, a set of clinical signs indicative of increased intracranial pressure. At this point, the patient did not appear to be in acute distress and would track with eyes and smile when the son spoke to her. She was moving her extremities but developed cogwheel rigidity bilaterally in the upper extremities.

**Table 1:** Table representing the basic metabolic panel of the patient. ↑: Increase from reference range, Mmol/L: millimoles per liter, Mg/dL: milligrams per deciliter, MEq/L: milliequivalents per liter.

Basic metabolic panel	Result	Reference range
Sodium (mmol/L)	163 (↑)	136-146
Potassium (mmol/L)	3.6	3.5-5.0
Chloride (mmol/L)	128 (↑)	98-106
Carbon dioxide(mmol/L)	23	24-31
Blood urea nitrogen (mg/dL)	11	6.0-20.0
Creatinine (mg/dL)	1.40 (↑)	0.50-0.90
Glucose (mg/dL)	131 (↑)	70-99
Calcium (mg/dL)	9.1	8.6-10.0
Anion gap (mEq/L)	12	5-15

**Table 2:** Table representing the complete blood workup of the patient. ↑: Increase from reference range, ↓: decrease from reference range, %: percentage in blood, % WBC: percentage in white blood cells, (K/uL): thousands per microliter, M/uL: millions per microliter, g/dL: grams per deciliter, fL: femtoliters, pg/cell: picograms per cell, WBC: white blood cell count, RBC: red blood cell count, HGB: hemoglobin, HCT: hematocrit, Abs: absolute count, MCV: mean corpuscular volume, MCH: mean corpuscular hemoglobin, MCHC: mean corpuscular hemoglobin concentration, MPV: mean platelet volume, RDW: red cell distribution width, PLT: platelet count, Imm Gran: immature granulocytes, NRBC: nucleated red blood cell.

CBC and differential of the patient component	Result	Ref range
WBC (K/uL)	20.38 (↑)	4.50-10.90
RBC (M/uL)	4.01	4.20-5.40
HGB (g/dL)	13	12.0-16.0
HCT%	42.3	370.-47.0%
MCV (fL)	105.5 (↑)	78.0-95.0
MCH (pg/cell)	32.4 (↑)	26.6-31.6
MCHC (g/dL)	30.7	30.5-35.5
MPV (fL)	10.8	6.4-12.2
RDW	12	11.5-15.1%
PLT (K/uL)	420	130-400
Neutrophil % WBC	90.3 (↑)	38.0-69.0%
Lymphocyte % WBC	1.4 (↓)	22.4-49.0%
Monocyte % WBC	7.6	2.4-9.2%
Eosinophil % WBC	0	0.0-8.6%
Basophil % WBC	0.1	0.0-1.0%
Imm Gran % WBC	0.6	0.00%
Neutrophil Abs (K/uL)	18.42 (↑)	1.51-7.30
Lymphocyte Abs (K/uL)	0.28 (↓)	0.88-5.93
Monocyte Abs (K/uL)	1.54 (↑)	0.09-1.11
Eosinophil Abs (K/uL)	0	0.00-1.04
Basophil Abs (K/uL)	0.02	0.00-0.10
Immature Gran Abs (K/uL)	0.12	0.00
NRBC Abs (K/uL)	0	<4.0

**Table 3:** Table representing the vital signs of the patient. bpm: beats per minute, mm Hg: millimeters of mercury, °F: degrees Fahrenheit.

Vital sign	Result	Reference range
Heart rate (bpm)	55	60–100
Respiratory rate (breaths per minute)	17	12–20
Blood pressure (mm Hg)	141/70	90–139/60-89
Temperature (°F)	98.6	97.9–100.4

**Figure 1:**
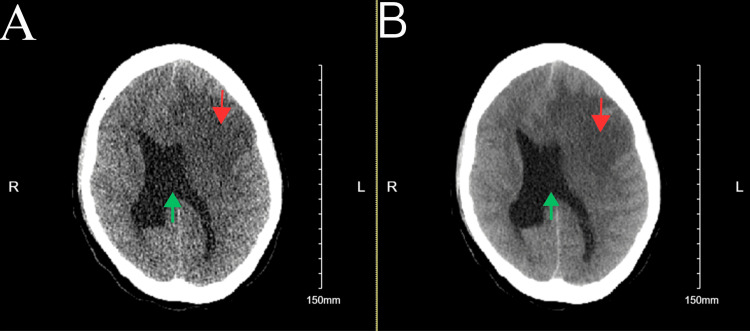
Computed tomography (CT) scan of the head without contrast demonstrated a large heterogeneous mass centered about the left frontal lobe with stable locoregional mass effect findings (red arrows); specifically persistent left-to-right midline shift of 9 millimeters (green arrows) with subfalcine and left uncal herniation. (A) Computed tomography scan without contrast exhibiting a more uniform grayscale and less differentiation between different tissue densities. (B) Computed tomography scan without contrast exhibiting more defined parenchymal structures and a greater differentiation between tissues. Red arrows: Demonstration of the large heterogeneous mass centered about the left frontal lobe with stable locoregional mass effect findings. Green arrows: Persistent left-to-right midline shift of 9 millimeters.

**Figure 2:**
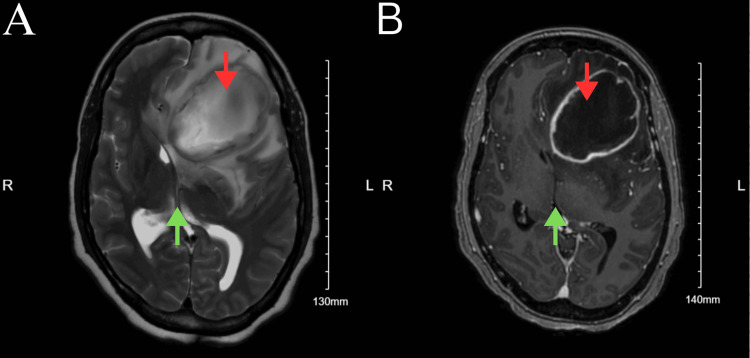
T2-weighted (A) and fluid-attenuated inversion recovery (FLAIR) (B) magnetic resonance imaging (MRI) demonstrating a left frontal lobe mass measuring 6.2 × 4.6 × 3.7 centimeters with thick enhancing wall and central non-enhancement (red arrows). Significant surrounding edema with 1.2-centimeter rightward midline shift (green arrows) and effacement of the left frontal horn. (A) T2-weighted magnetic resonance imaging (MRI), (B) fluid-attenuated inversion recovery (FLAIR) magnetic resonance imaging (MRI). Red arrows: Left frontal lobe mass measuring 6.2 × 4.6 × 3.7 centimeters with thick enhancing wall and central non-enhancement effacement of the left frontal horn. Green arrows: 1.2-centimeter rightward midline shift.

**Figure 3:**
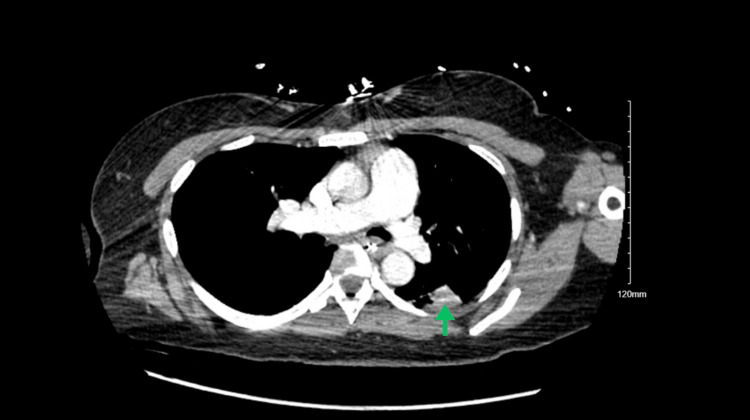
Computed tomography (CT) scan with contrast demonstrating a left basilar pulmonary arteriovenous malformation measuring up to 2 cm. Green arrow: Left basilar pulmonary arteriovenous malformation.

Due to altered mental status and potential increase of intracranial pressure, a delirium workup was initiated, and a non-contrast computed tomography (CT) was completed on March 8, 2024, at 5:29 p.m. The CT scan demonstrated a large heterogeneous mass centered about the left frontal lobe with a stable locoregional mass effect and associated moderate vasogenic edema. A persistent left-to-right midline shift of 9 mm with subfalcine and left uncal herniation was also revealed, along with the internal development of right frontal subarachnoid hemorrhage. CT scan also demonstrated right and left lateral and third ventricle effacement (Figure 1). A magnetic resonance imaging (MRI) was also completed, which showed a 6.2 × 4.6 × 3.7 cm sized left frontal lesion suspicious for abscess due to central restricted diffusion and obstructive hydrocephalus with dilatation of the temporal horn of the right lateral ventricle (Figure 2). These findings were more indicative of an abscess compared to malignancy. A CT scan of the chest was also completed on March 9, 2024, which found an arteriovenous malformation (AVM) at the left posterior lung base and pulmonary artery, likely to be the precipitating cause leading to the brain abscess (Figure 3). By the end of the day, the patient's condition significantly deteriorated; she was no longer responsive and exhibited asymmetric pupils with bilateral myoclonic jerking movements. This warranted emergent intubation, scheduling of surgical aspiration of the cerebral abscess, and administration of dexamethasone 10 mg and mannitol due to concerns regarding herniation. A left hemicraniectomy and aspiration of cerebral abscess procedure was completed on March 9, 2024, at 6:00 p.m. Large amounts of purulent fluid were removed outside of the cystic area from the left interior frontal region, and the cyst was aspirated, containing a large amount of purulent fluid. The purulent fluid was sent for culture, which was later revealed to be positive for *Streptococcus viridans*. The patient was recovering well from the surgery and exhibited more sustained eye-opening the following week. 

**Figure 4:**
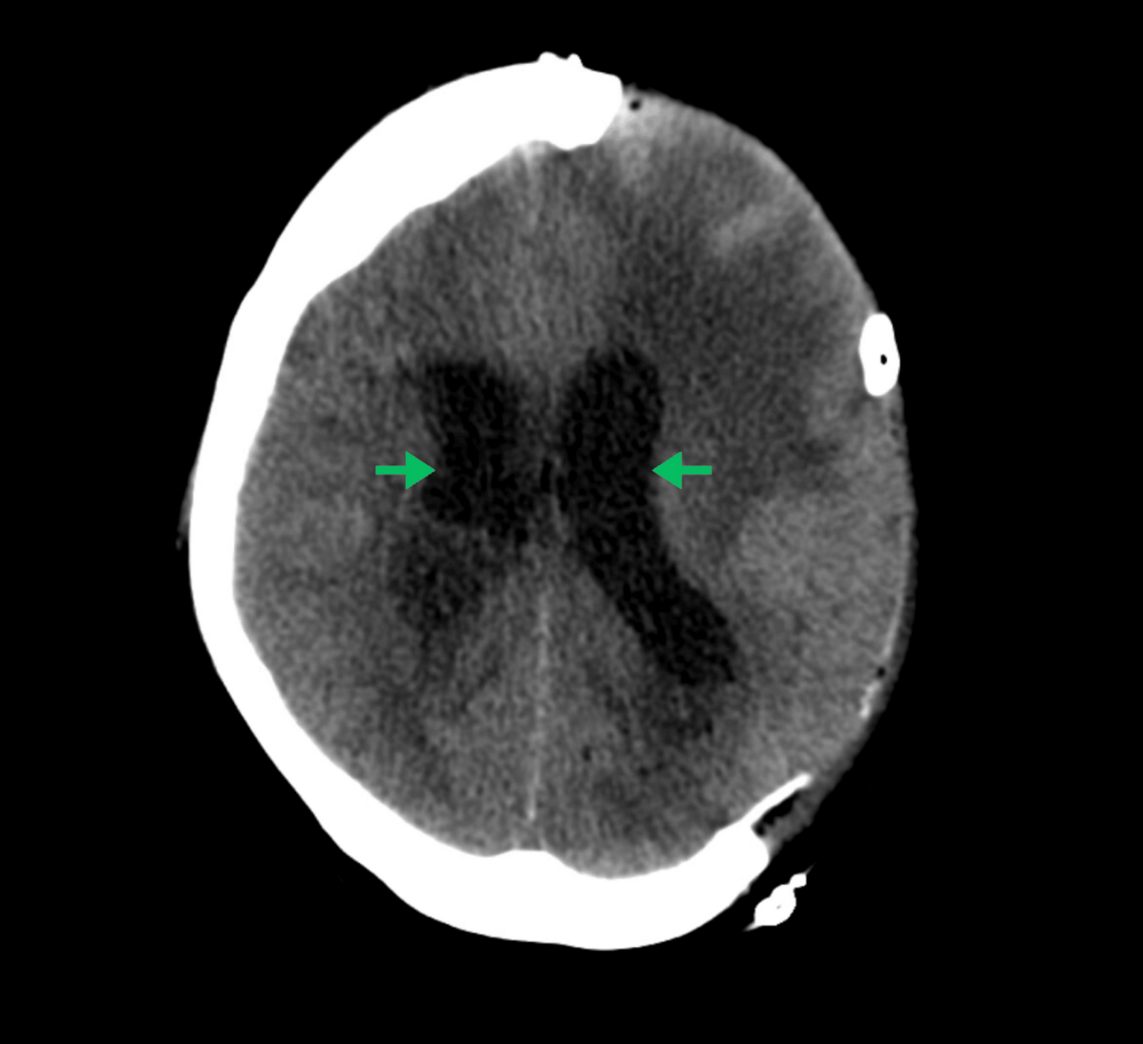
Computed tomography (CT) of the head without contrast demonstrating moderate dilatation of the lateral ventricles. Green arrows: Bilateral dilatation of the lateral ventricles.

On March 11, 2024, a head CT demonstrated an interval increase in the size of ventricles that was indicative of communicating hydrocephalus, presumably from ventriculitis (Figure 4). This led to a ventricular drain placement and numerous failed clamp trials. The patient also had completed a left pulmonary artery angiogram with selective and superselective left pulmonary artery branch catheterization and embolization on March 22, 2024, for the AVM visualized by CT upon admission (Figure 4). Due to the persistent hydrocephalus, her family agreed to the neurosurgical placement of a right ventricular peritoneal shunt. This procedure was completed on April 9, 2024, with the strata valve set to 1.5 and CSF fluid collected for analysis. During recovery, the patient had a witnessed seizure that lasted two minutes that self-resolved and was treated with lorazepam and levetiracetam, followed by a brief episode of tachycardia with a heart rate in the 190 bpm range. The patient remained awake and alert throughout recovery with a tracheostomy tube placed and surgical incisions healing well. Pupils were sluggish but symmetrical and the patient was able to regard staff and visitors. Prior to discharge, she was able to follow simple commands, squeeze both hands, and move the left upper and lower extremities spontaneously, but she had minimal spontaneous movement of the right upper and lower extremities. She was subsequently placed on discharge to a nearby subacute rehab center on May 5, 2024.

## Discussion

Diagnosing and treating cerebral brain abscesses, especially in immunocompromised persons like HIV-positive patients, present considerable challenges. The current case illustrates the importance involved in recognizing a brain abscess that can mimic and display symptoms of psychiatric disorders, emphasizing the utility of diagnostic assessments and maintaining a high level of suspicion in similar patients.

Clinical presentation and diagnostic challenges

Because clinical symptoms of brain masses depend on numerous factors, such as the size and location of the lesion [3], it is inherently difficult to diagnose brain abscesses based on symptomatology. Our case demonstrates how a clinical diagnosis can be further complicated by the presentation of psychiatric symptoms such as catatonia and disorganized behavior, especially in a patient with a past psychiatric history. Psychiatric symptoms such as catatonia may make it difficult to diagnose underlying physical diseases in a timely manner. Catatonia is a unique illness with neurological underpinnings that may be overlooked and instead attributed to psychiatric manifestations [3]. The patient's rapid development of medical and neurological symptoms, including cogwheel rigidity, myoclonic jerking, asymmetric pupils, hypertension, and hypernatremia, prompted the investigation of an organic etiology, leading to the use of confirmatory neuroimaging.

The presence of an arteriovenous malformation (AVM) in the lungs suggests that the brain abscess, in this case, was caused by the transmission of infection through the bloodstream. This is consistent with processes by which systemic illnesses can spread to the brain, especially in individuals with HIV and other diseases, leading to an immunocompromised state [4]. The presence of HIV significantly increases the likelihood of such infections because it weakens the immune system's ability to detect and respond to them.

Although rare, there have been some cases presented in the academic literature that similarly demonstrate how brain abscesses can present with psychiatric symptoms. In one example, D’agostino et al. reported on a patient with a subdural abscess with symptoms of severe depression with suicide ideation. The abscess being a causing factor was only revealed once an MRI was completed due to a prior history of a craniotomy [5]. Similarly, the assumption of a psychiatric etiology based on symptoms led to the delay of diagnosing the underlying infectious process, which signifies the need to add organic causes to the differential diagnosis early in patients with new or atypical psychiatric symptoms. This is particularly important to consider before worsening neurological or medical presentations that may occur.

Imaging, management and treatment outcomes

Neuroimaging plays a vital role in the diagnosis of brain abscesses. In our case, the initial non-contrast CT scan detected a heterogeneous mass with surrounding edema and a midline displacement, which required additional investigation using MRI. The MRI scan yielded more detailed imaging, indicating the presence of an abscess rather than a malignant tumor. This underscores the importance of utilizing advanced imaging techniques when evaluating potential organic causes, even if the presentation may appear to be psychiatric. Neuroimaging is crucial in detecting intracranial infections, which may exhibit unusual symptoms such as profound depression or suicidal ideations [5].

Fukui et al. documented a case in which a subdural abscess initially manifested with depressive symptoms and was treated with antibiotics alone once imaging confirmed the lesion [6]. Advanced imaging modalities were crucial for precise diagnosis in this case and ours. However, the patient we presented required immediate neurosurgical intervention due to rapidly declining status. The comparison of these instances reaffirms the necessity of neuroimaging in investigating psychiatric symptoms resulting from underlying infectious processes rather than main psychiatric diseases and the appropriate interventions necessary, which depend on the particular case and severity.

The management of this condition requires a comprehensive strategy that includes multiple disciplines, such as neurology, neurosurgery, psychiatry, and medicine. The patient underwent a left hemicraniectomy and abscess aspiration, which were essential in treating the infection and decreasing pressure inside the skull. The progression of hydrocephalus necessitated other medical procedures, such as the insertion of a ventricular drain and a ventriculoperitoneal shunt. In our patient, an efficient surgical and medical intervention that was implemented within two days of admission was essential for preventing further neurological deterioration. D’agostino et al. note the necessity for immediate surgical and pharmacological intervention within 21 hours of admission, leading to the effective management of the epidural abscess [5]. Another case report demonstrated a patient who had cataplexy, mutism, and unprovoked agitation for 20 days prior to recognition of a massive epidural abscess, which was only identified due to a lack of response after the increase of psychiatric medication was ineffective. Despite the recognition of urgent neurosurgical intervention once visualized, she succumbed to her illness prior to surgery [3]. This highlights the need to promptly include organic causes and incorporate neuroimaging accordingly for patients with worsening symptoms that may be treatment-resistant or new onset psychiatric conditions that were never present in past evaluations.

Delirium is a complex neuropsychiatric syndrome observed in patients with underlying neurological conditions, particularly those with altered mental status. The management of delirium often requires addressing both the patient's acute symptoms and the underlying causes that are contributing to the patient's delirium. In our case, levetiracetam and lorazepam were used for the management of catatonia and the witnessed seizure that occurred during recovery. While benzodiazepines such as lorazepam are commonly used for the management of both seizures and catatonia, caution is warranted due to the risk of exacerbating delirium. When using benzodiazepines, it is important to consider the risk-benefit ratio and evaluate factors such as age, hospitalization status, and previous benzodiazepine use if delirium is suspected [7]. In our case report, the altered mental status and catatonia were primarily attributed to an organic cause of brain abscess, which was effectively managed through neurosurgical interventions. As a result, careful consideration was given before determining that lorazepam was an appropriate and safe option for treatment.

Psychiatric implications

The psychiatric symptoms associated with brain abscesses, as observed in this instance, emphasize the complex connection between infections in the central nervous system and psychiatric illnesses. Symptoms such as catatonia, disorganized behavior, anxiety, and psychosis must not always be considered products of primary psychiatric disorders, and less common organic causes must be appropriately ruled out in a timely manner. It is important to explore whether individuals are diagnosed with brain abscesses and their relation to experiencing psychiatric symptoms such as depression, mania, delusions, or catatonia. Although our patient had a past psychiatric history of PTSD and bipolar disorder, she never exhibited disorganized behavior, tremulousness, or catatonia in the past. The unusual nonadherence to therapy and medication, disorganized speech, and novel vocal cues were the factors that led to the outpatient psychiatrist's concern and organization of the home visit. This demonstrates the necessity for mental health providers to develop a cohesive understanding of their patients’ presentations, allowing them to discern subtle deviations from baseline status. This awareness is crucial in identifying novel occurrences that may be erroneously attributed to an existing diagnosis. Combining rigorous mental and medical care is necessary to effectively manage intricate neuroinfectious illnesses that require management in a timely and effective manner.

It is also imperative to assess the relationship between patients with a brain abscess and how it may affect the treatment of comorbid psychiatric disorders. Research conducted by Omland et al. investigated the mental health consequences in patients who were diagnosed with brain abscesses. The study revealed a notable rise in the utilization of psychiatric drugs and outpatient treatment after the diagnosis [8]. The heightened susceptibility to psychiatric illnesses in individuals with brain abscesses necessitates diligent surveillance and comprehensive treatment to manage both neurological and psychiatric manifestations effectively. This is especially true when managing patients who have existing psychiatric disorders that may still exhibit symptoms from the abscess even after appropriate treatment. This may hinder the ability for compliance to medication or other psychiatric assistance that may be necessary. The employment of a multidisciplinary strategy is necessary to effectively manage patients with both psychiatric and infectious conditions, ensuring that both components are thoroughly handled.

## Conclusions

This case highlights the importance of comprehensive diagnostic assessments and the consideration of a brain abscess or other organic causes in individuals who display rare, atypical psychiatric symptoms such as catatonia, especially in those who are immunocompromised. Neuroimaging techniques and adopting a multidisciplinary therapeutic approach are imperative for properly controlling brain abscesses. To achieve the best outcomes for patients, it is necessary to have a high level of suspicion and collaborative treatment due to the complex relationship between psychiatric and neurological symptoms that a brain abscess may present with.
